# Dopamine Modulates Excitatory Synaptic Transmission by Activating Presynaptic D1-like Dopamine Receptors in the RA Projection Neurons of Zebra Finches

**DOI:** 10.3389/fncel.2020.00126

**Published:** 2020-05-12

**Authors:** Songhua Wang, Shaoyi Liu, Qingqin Wang, Yalun Sun, Lihua Yao, Dongfeng Li, Wei Meng

**Affiliations:** ^1^Jiangxi Key Laboratory of Organic Chemistry, Jiangxi Science and Technology Normal University, Nanchang, China; ^2^School of Life Science, South China Normal University, Guangzhou, China; ^3^School of Life Science, Jiangxi Science and Technology Normal University, Nanchang, China

**Keywords:** dopamine, excitatory synaptic transmission, D1-like dopamine receptors, the robust nucleus of the arcopallium, zebra finch

## Abstract

Songbirds are useful vertebrate study models for vocal learning and memory. The robust nucleus of the arcopallium (RA) receives synaptic inputs from both the posterior and anterior pathways of the song control system in songbirds. Hence, RA plays an important role in the control of singing. RA receives dopaminergic (DArgic) inputs that increase the excitability of RA projection neurons (PNs). However, the effects of DA on excitatory synaptic transmission are yet to be deciphered. In this study, the effects of DA on the excitatory synaptic transmission of the PNs in the RA of adult male zebra finches were investigated using a whole-cell patch-clamp recording. We observed that DA decreased the frequency of spontaneous excitatory postsynaptic currents (sEPSCs) and miniature excitatory postsynaptic currents (mEPSCs). The effects of DA were mimicked by the D1-like DA receptor (D1R) agonist, SKF-38393, but not the D2-like DA receptor (D2R) agonist, Quinpirole. Also, the effects of DA were blocked by D1R antagonist, SCH-23390, but not the D2R antagonist, Sulpiride. These results demonstrate that DA modulates excitatory synaptic transmission by acting on D1R in the RA of adult male zebra finches.

## Introduction

**Figure 1:**
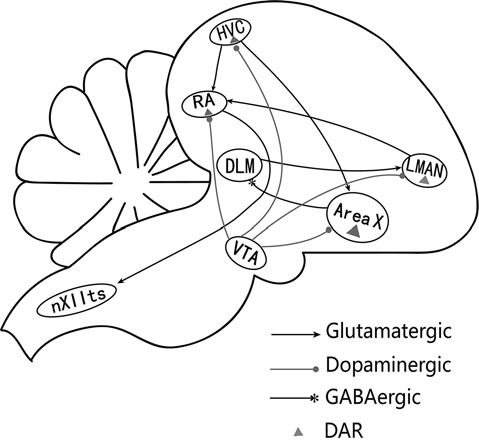
A simplified diagram of the song control system (Nottebohm et al., 1976). The song system consists of two major pathways. The vocal motor pathway (VMP) starts with nucleus HVC (letter-based name). HVC projects to the robust nucleus of archistriatum (RA), which innervates the tracheosyringeal part of the hypoglossal nucleus (nXIIts). The anterior forebrain pathway (AFP) starts with the projection from HVC to Area X, AreaX projects to the medial portion of the dorsolateral nucleus of the anterior thalamus (DLM), which sends its output to the lateral magnocellular nucleus of the anterior nidopallium (LMAN), then projects to RA (Ding et al., 2003). RA receives glutamatergic inputs from HVC and LMAN (Mooney and Konishi, 1991). HVC, RA, Area X, and LMAN also receive dopaminergic inputs from the ventral tegmental area (VTA) and express dopamine receptors (Kubikova and Kostál, 2010).

Birdsong is a complex learned behavior in Avians. Learning and song production is mediated by a discrete set of interconnected brain nuclei, i.e., the song control system (Nottebohm et al., 1976). The song system can be divided into two pathways (Figure 1): the vocal motor pathway (VMP) that contributes to song production and the anterior forebrain pathway (AFP) that is essential for song learning and plasticity (Brainard and Doupe, 2000). The robust nucleus of the arcopallium (RA) is the key site that receives afferent inputs from both the HVC (letter-based name) and lateral magnocellular nucleus of the anterior nidopallium (LMAN; Mooney and Konishi, 1991). RA activity is significantly correlated with acoustic features of syllables (Sober et al., 2008), and is important for accurate timing and structural bursts of activity associated with specific syllables (Yu and Margoliash, 1996). The RA consists of projection neurons (PNs) and inter-neurons that receive excitatory glutamatergic inputs from the HVC and LMAN. However, they have distinct postsynaptic properties. The HVC-RA input is primarily mediated through the α-amino-3-hydroxy-5-methyl-4-isoxazolepropionic acid receptor (AMPAR), whereas the LMAN input to RA is primarily mediated through the N-methyl-D-aspartic acid receptor (NMDAR; Mooney and Konishi, 1991). Also, PNs receive glutamatergic inputs from the axon collateral of other RA PNs and GABAergic inputs from RA interneurons (Sizemore and Perkel, 2008).

The dopaminergic (DArgic) system participates in several cognitive and behavioral activities which include reward, motor control, learning and memory in both mammals and songbirds (Gadagkar et al., 2016; Hoffmann et al., 2016). Dopamine modulates human speech and bird song control (Simonyan et al., 2012). When male songbirds sing to females (directed song), the DA levels in Area X increase (Sasaki et al., 2006). Also, DA can directly modulate intrinsic electrophysiological properties and synaptic inputs of Area X (Ding and Perkel, 2002; Budzillo et al., 2017). RA expresses both D1 (D1R) and D2 receptors (D2R) and receives DArgic inputs (Kubikova and Kostál, 2010). However, the function of these projections is yet to be deciphered. Understanding how the activation of DA could affect synaptic activity in the RA will provide a mechanistic understanding of the neural control of song behavior.

Our previous study demonstrated that DA could modulate the excitability of RA PNs (Liao et al., 2013) and D1R could increase the NMDA-induced gain modulation (Wang et al., 2015a). DA has been demonstrated to be involved in adult song plasticity. How DA affects synapse input to the RA is unknown. In this study, we examined the effects of DA on excitatory synaptic transmission to RA PNs using the whole-cell patch-clamp technique.

## Materials and Methods

### Preparation of Brain Slices

All experimental protocols were approved by the Institutional Animal Care and Use Committee of Jiangxi Science and Technology Normal University. Coronal brain slices (250 μm thick) were generated from adult male zebra finches (>90 days old) as previously described (Meng et al., 2017; Wang et al., 2019). Briefly, birds were anesthetized with 10% chloral hydrate and then beheaded. The brain was exposed and then brain slices were prepared using a vibrating microtome (700 ms; Campden Instruments, UK). The brain was dissected in ice-cold, oxygenated (95% O_2_ and 5% CO_2_) slice solution. Slice solution was composed of (in mM) sucrose 248, KCl 5, NaHCO_3_ 28, glucose 10, MgSO_4_ · 7H_2_O 1.3, and NaH_2_PO_4_ · H_2_O 1.26. Slices were incubated in artificial cerebrospinal fluid (ACSF) at 37°C. ACSF was composed of (in mM) NaCl 125, KCl 2.5, NaH_2_PO_4_ · H_2_O 1.27, MgSO_4_ · 7H_2_O 1.2, NaHCO_3_ 25, glucose 25, and CaCl_2_ 2.0. Osmolality was adjusted using sucrose to 350 mOsm (Meng et al., 2017). Slices were incubated in a holding chamber for at least 1.5 h and equilibrated to room temperature before patch-clamp recordings.

### Patch-Clamp Recordings

Electrophysiological recordings were performed at room temperature (24–28°C) using oxygenated ACSF. RA and the surrounding tissues were observed using a BX51WI microscope with a DIC-IR video camera (Olympus, Tokyo, Japan). Recording pipettes were fabricated from borosilicate glass (Sutter Instruments, Novato, CA, USA) using a Flaming-Brown puller (P-97, Sutter Instruments) and then filled with a solution containing (in mM): KMeSO_4_ 120, NaCl 5, HEPES 10, EGTA 2, QX-314 5, ATP 2, and GTP 0.3 (pH 7.2–7.4, 340 mOsm). The recording pipettes (with a resistance of 4–7 MΩ) were positioned using an integrated motorized control system (Sutter Instruments). Cell-attached and whole-cell recordings were performed using standard techniques. The two cell types in the RA, PNs and GABAergic interneurons, were identified based on their distinct electrophysiological properties(Spiro et al., 1999; Liao et al., 2011). PNs display regular spontaneous firing and generate a time-dependent inward rectification while hyperpolarized using current injection, while interneurons lack spontaneous firing in the resting state (Spiro et al., 1999; Liao et al., 2011). Brain slices were used only once after drug administration for recordings. The junction potentials were corrected before clamping the neurons. Pipette capacitance and series resistance were compensated online using MultiClamp 700B (Molecular Devices, Foster City, CA, USA). Series resistance was monitored at 2 min intervals. Signals were amplified and filtered with the MultiClamp 700B amplifier (low-pass filtered at 10 kHz). The recordings that showed series resistance >20 MΩ or 10% change were excluded from the analysis. Spontaneous excitatory postsynaptic currents (sEPSCs) were isolated by bath application of 150 μM picrotoxin (PTX) to block GABA receptor-mediated inhibitory synaptic currents. For miniature excitatory postsynaptic currents (mEPSCs) recordings, 1 μM tetrodotoxin (TTX) was added into the bath in addition to PTX. The recorded neurons were allowed to stabilize for 3–5 min after the rupturing of the patch. sEPSCs/mEPSCs were recorded for 10–15 min while the membrane potential was held at −70 mV throughout the whole-cell recording. Signals were amplified using MultiClamp 700B.

### Drug Application

Bath perfusate containing the standard ACSF was changed to ACSF containing the various drugs. The bath was continuously mixed and oxygenated with 95% O_2_ and 5% CO_2_. The following pharmacological reagents were used: DA (100 μM), SKF-38393 (10 μM), SCH-23390 (20 μM); quinpirole (10 μM) and sulpiride (10 μM; Ding et al., 2003; Liao et al., 2013). All drugs were obtained from Sigma–Aldrich (St. Louis, MO, USA).

### Data Analysis

Data were obtained using Clampfit 10.5 (Molecular Devices, San Jose, CA, USA) *via* a DIGIDATA 1550B series A/D board (Molecular Devices, San Jose, CA, USA) at a sampling frequency of 10 kHz. Data from sEPSCs and mEPSCs were analyzed using Clampfit 10.5 (Molecular Devices, San Jose, CA, USA), Mini 6.0 (Synaptosoft Inc., Fort Lee, NJ, USA) and Origin Pro 8.0 (OriginLab, Northampton, MA, USA) on a PC. Average values of inter-event interval (IEI) and amplitude for events in the control and drug administered group were compared. The mean of the averaged values for each group was then statistically compared using paired *t*-test unless otherwise stated. Cumulative probability distributions of IEI and amplitude were assessed to determine the significance of the shifts using the nonparametric Kolmogorov–Smirnoff (K-S) test. To analyze the kinetic properties of the sEPSCs/mEPSCs, 100 s EPSCs’ recordings for each cell were selected and the EPSCs in the time course were averaged. Approximately 100–500 events were recorded for each cell. The decay of the average current was fitted using a mono-exponential function with a single decay time constant. The rise time was defined as the average current rise time from 10% to 90% of the peak (Simonyan et al., 2012). All data were presented as mean ± SEM unless otherwise stated (Wang et al., 2015b).

## Results

Stable whole-cell recordings were obtained using 112 RA PNs from 40 zebra finches.

### DA Decreases the Frequency But Not the Amplitude of sEPSCs in RA PNs

**Figure 2:**
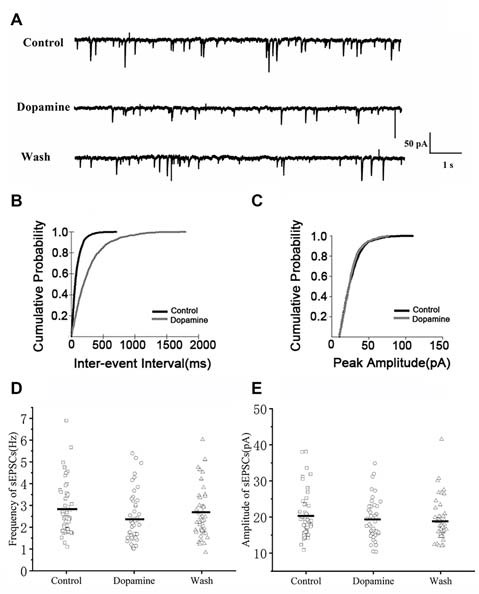
Effects of dopamine (DA) on spontaneous excitatory postsynaptic currents (sEPSCs) in arcopallium (RA) projection neurons (PNs). **(A)** Representative sample traces of sEPSCs recordings for control and DA treatment. **(B)** Cumulative inter-event interval (IEI) distributions for sEPSCs demonstrating that DA could increase intervals (*p* < 0.01). **(C)** Cumulative amplitude distributions for sEPSCs demonstrating that DA did not affect the amplitude of sEPSCs (*p* > 0.05). **(D,E)** DA significantly reduced the frequency but not the amplitude in RA PNs. Horizontal lines indicate median values.

To investigate the effects of DA (100 μM) on glutamatergic transmission, sEPSCs were recorded from RA PNs. For DA-treated slices, the frequencies of sEPSCs were significantly lower compared to the control (Figures 2A,D). Cumulative probability plots of IEI are shown in Figure 2B. DA increased the proportion of longer IEIs. The frequency of sEPSCs was 2.81 ± 0.17 Hz in the control and 2.41 ± 0.16 Hz after 5 min of adding DA (*n* = 48; *p* < 0.01; Figure 2D), and returned to the baseline after washing (Wash: 2.64 ± 0.17 Hz). Cumulative probability plots in Figure 2C show the amplitudes of the sEPSCs recordings for both the control and DA-treated slices. DA did not affect the distribution of the amplitudes. The amplitude of sEPSCs after DA addition did not change from the control (control: 20.37 ± 0.96 pA; DA: 19.23 ± 0.78 pA; *p* > 0.05, wash: 19.10 ± 0.82 pA; *n* = 48; Figure 2E). Also, the rise and decay times of the sEPSCs were not affected by DA (Table 1).

**Table 1:** Effects of DA on the rise and decay time of spontaneous excitatory postsynaptic currents (sEPSCs) and miniature excitatory postsynaptic currents (mEPSCs).

	*n*	Rise time (ms)		Decay time (ms)	
		Control	DA	Control	DA
sEPSC	48	4.01 ± 0.12	4.07 ± 0.10 (NS)	6.25 ± 0.22	6.40 ± 0.27 (NS)
mEPSC	28	3.55 ± 0.09	3.79 ± 0.10 (NS)	5.36 ± 0.30	5.43 ± 0.24 (NS)

*Values are presented as mean ± SEM. n denotes the number of cells; NS denotes not significant. We selected 120 s recording samples of sEPSCs/mEPSCs for each cell and averaged the sEPSCs/mEPSCs during the time course. The rise and decay times constants of sEPSCs and mEPSCs recorded from RA PNs were not significantly altered in the presence of DA*.

### DA Reduces the Frequency But Not the Amplitude of mEPSCs in RA PNs

**Figure 3:**
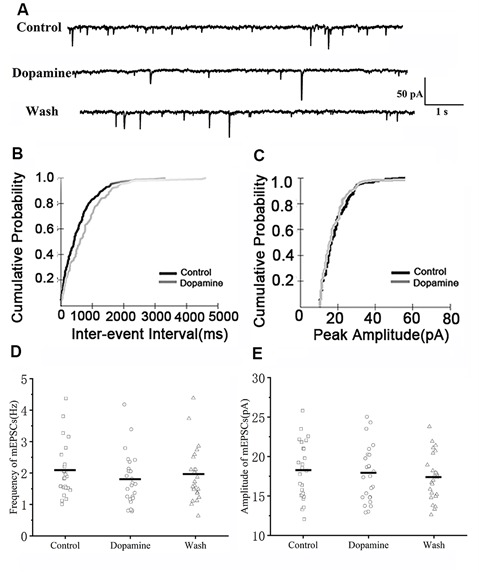
Effects of DA on miniature excitatory postsynaptic currents (mEPSCs) in RA PNs. **(A)** Representative sample traces of mEPSCs recording from RA PNs before and after DA addition to tissue slices. **(B)** Cumulative IEI distribution for mEPSCs for control and DA treated samples. **(C)** Cumulative amplitude distributions of mEPSCs in the control and DA treated samples. DA notably increased the interval between mEPSCs events (*p* < 0.01) but did not affect their amplitudes (*p* > 0.05). **(D,E)** DA significantly reduced the frequency but not the amplitude in RA PNs. Horizontal lines indicate median values.

To determine whether DA acts only on presynaptic termini or also on the postsynaptic component, we examined the effects of DA (100 μM) on mEPSCs. This was because mEPSCs are believed to be the postsynaptic response to a single spontaneously released synaptic vesicle (Yao et al., 2013). Changes in the frequency of mEPSCs are thought to result from presynaptic components, while changes in the amplitude of mEPSCs indicate alterations in postsynaptic components (Hsia et al., 1998; Regehr and Stevens, 2001; Cooke and Woolley, 2005). Similar to the results obtained for sEPSCs, sample traces observed for mEPSCs are shown in Figure 3A. Cumulative probability plots of IEI are shown in Figure 3B. DA increased the proportion of longer IEIs. Cumulative probability plots in Figure 3C show the amplitude of mEPSCs recordings for both the control and DA-treated slices. This demonstrated that DA had no effect on the distribution of the amplitudes. The dot graphs in Figures 2E, 3D present the mean of frequency and amplitude. DA decreased mEPSCs frequency from 2.07 ± 0.17 Hz to 1.78 ± 0.17 Hz (*n* = 25; *p* < 0.01; Figure 3D) and returned to the baseline after washing (Wash: 1.93 ± 0.15 Hz) however, the amplitude of the mEPSCs was not altered by DA (control: 18.19 ± 0.73 pA; DA: 17.77 ± 0.71 pA; *p* > 0.05, wash: 17.37 ± 0.51 pA; *n* = 25; Figure 3E). The rise and decay times of the mEPSCs did not change in the presence of DA, and were similar to sEPSCs (Table 1).

### DA Inhibits Excitatory Synaptic Transmission *via* D1R

Both D1R and D2R are expressed in the RA (Kubikova and Kostál, 2010), thus either subtype could mediate the synaptic actions of DA. To elucidate the receptor subtype mediating DA-induced reductions in mEPSCs, the effects of specific DA receptor agonists and antagonists were investigated.

**Figure 4:**
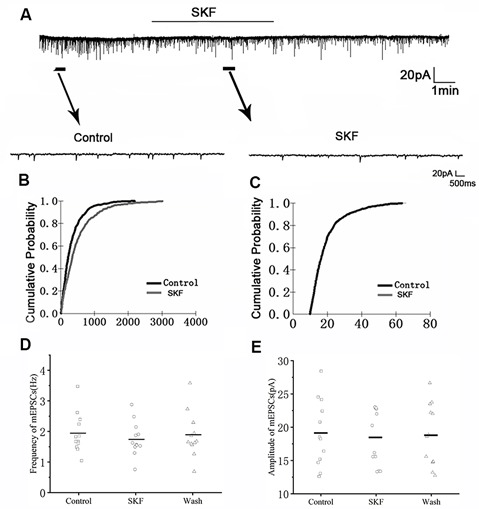
Effects of D1-like DA receptor (D1R) agonist SKF-38393 on mEPSCs in RA PNs. **(A)** Recordings of mEPSCs in the absence and presence of SKF38393. **(B)** Cumulative IEI distributions for mEPSCs in the control and after application of SKF (*p* < 0.01). **(C)** Cumulative amplitude distributions of mEPSCs in the control and after application of SKF. **(D,E)** SKF significantly decreased the frequency, but not the amplitude of mEPSCs in RA PNs. Horizontal lines indicate median values.

**Figure 5:**
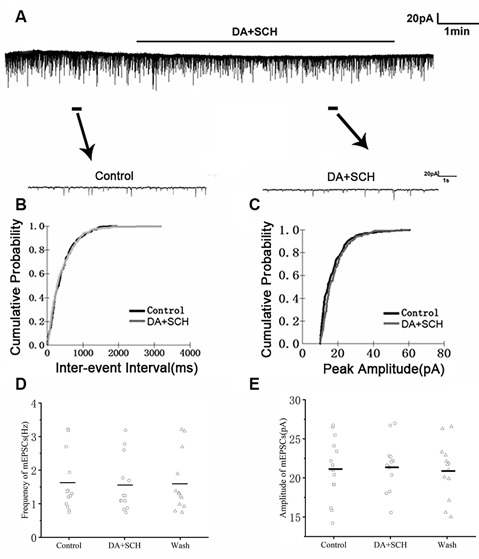
Effects of DA and D1R antagonist SCH-23390 (20 μM) on mEPSCs in RA PNs. **(A)** Recordings of mEPSCs in the absence and presence of SCH and DA. **(B)** Cumulative IEI distributions for mEPSCs in the control and after DA and SCH (*p* > 0.05). **(C)** Cumulative amplitude distributions of mEPSCs in control and after DA and SCH (*p* > 0.05). **(D,E)** DA and SCH did not affect the frequency and amplitude of mEPSCs in RA PNs. Horizontal lines indicate median values.

We first examined the effect of D1R in RA PNs using the D1R agonist SKF-38393 (10 μM). Sample traces of mEPSCs are shown in Figure 4A. Cumulative probability plots of IEI are shown in Figure 4B. SKF increased the proportion of longer IEIs. Cumulative probability plots in Figure 4C show the amplitude of mEPSCs recordings for both the control and SKF-treated slices. SKF decreased the frequency of mEPSCs (from 1.94 ± 0.19 Hz to 1.76 ± 0.16 Hz, *p* < 0.01, wash: 1.87 ± 0.21 Hz; *n* = 12; Figure 4D). However, the amplitude of the mEPSCs was not altered by SKF (control: 19.08 ± 1.45 pA; SKF: 18.44 ± 1.21 pA; wash: 18.89 ± 1.35 pA; *n* = 12; *p* > 0.05; Figure 4E). We then used DA and the D1R antagonist SCH-23390 (20 μM) to investigate whether D1R contributes to the effect of DA. Sample traces of mEPSCs are shown in Figure 5A. Cumulative probability plots of IEIs and amplitudes are shown in Figures 5B,C. DA and SCH did not affect the distribution of IEI and amplitudes. DA and SCH had no effect on mEPSCs frequency (control: 1.61 ± 0.24 Hz; DA + SCH: 1.56 ± 0.22 Hz, *p* > 0.05, wash: 1.60 ± 0.24 Hz; *n* = 13; Figure 5D) and amplitude (control: 21.29 ± 1.13 pA; DA + SCH: 21.53 ± 0.89 pA; *p* > 0.05, wash: 21.03 ± 0.99 pA; *n* = 13; Figure 5E). These results indicate that D1R contributes to the inhibitory effect of DA on the mEPSCs of RA PNs.

**Figure 6:**
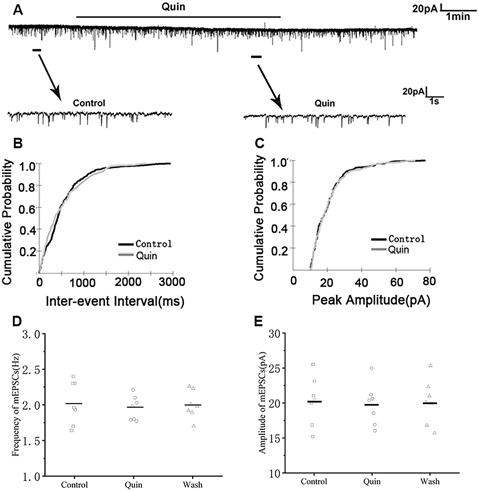
Effects of D2-like DA receptor (D2R) agonist quinpirole on mEPSCs in RA PNs. **(A)** Recordings of mEPSCs in the absence and presence of quinpirole. **(B)** Cumulative IEI distributions for mEPSC in the control and after addition of quinpirole *(p* > 0.05). **(C)** Cumulative amplitude distributions of mEPSCs in the control and after addition of quinpirole (*p* > 0.05). **(D,E)** Quinpirole did not affect the frequency and amplitude of mEPSCs in RA PNs. Horizontal lines indicate median values.

**Figure 7:**
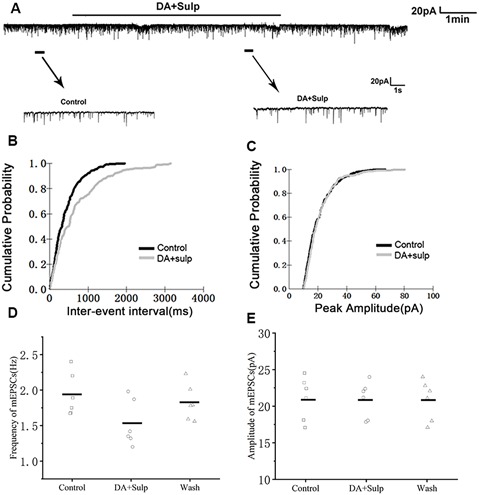
Effects of DA and D2R blocker, sulpiride, on mEPSCs in RA PNs. **(A)** Recordings of mEPSCs in the absence and presence of sulpiride and DA. **(B)** Cumulative distributions of IEIs for mEPSCs in the control and after addition of DA and sulpiride (*p* < 0.01). **(C)** Cumulative distributions of the amplitudes for mEPSCs in the control and after addition of DA and sulpiride (*p* > 0.05). **(D,E)** DA and sulpiride significantly decreased the frequency but not the amplitude of mEPSCs in RA PNs. Horizontal lines indicate median values.

We then used the D2R agonist quinpirole (10 μM) to determine whether D2R was involved in the effect of DA. Sample traces of mEPSCs are shown in Figure 6A. Cumulative probability plots of IEIs and amplitudes are shown in Figures 6B,C. Quinpirole did not affect the distribution of IEIs and amplitudes. Quinpirole had no effect on mEPSCs frequency (control: 2.03 ± 0.18 Hz; Quinpirole: 1.95 ± 0.22 Hz; *p* > 0.05, wash: 2.00 ± 0.23 Hz; *n* = 7; Figure 6D). The amplitude of the mEPSCs were also not altered by quinpirole (control: 20.30 ± 1.05 pA; quinpirole: 19.80 ± 0.93 pA; *p* > 0.05, wash: 20.15 ± 0.97 pA; *n* = 7; Figure 6E). Also, we used DA and D2R antagonist sulpiride (10 μM) to examine these effects. Sample traces of mEPSCs are shown in Figure 7A. Cumulative probability plots of IEIs are shown in Figure 7B. DA and sulpiride increased the proportion of longer IEIs. Cumulative probability plots in Figure 7C show the amplitude of mEPSCs recordings for both the control, DA and sulpiride-treated slices. DA and sulpiride did not affect the distribution of the amplitudes. DA and sulpiride decreased mEPSCs^’^ frequency (from 1.93 ± 0.08 Hz to 1.52 ± 0.09 Hz, *p* < 0.01, wash: 1.83 ± 0.07 Hz *n* = 6; Figure 7D) but not the amplitude (control: 21.06 ± 0.81 pA; DA + sulpiride: 20.91 ± 0.69 pA; *p* > 0.05, wash: 20.84 ± 0.76 pA *n* = 6; Figure 7E). These results indicated that D2R was not involved in the inhibitory effects of DA on the mEPSCs of RA PNs.

## Discussion

We demonstrated that DA reduced excitatory synaptic transmissions in RA PNs. DA robustly decreased the frequency of sEPSCs but did not affect its amplitude. This suggested that the functional synaptic transmission between neurons is significantly modulated by DA (Tian et al., 2012). We then evaluated mEPSCs to determine whether DA had presynaptic or postsynaptic effects. We observed that DA decreased the frequency but not the amplitude of mEPSCs, suggesting that DA acts on presynaptic sites to inhibit glutamate release (Basavarajappa et al., 2008). These results are similar to those obtained in rat nucleus tractus solitarius (Ohi et al., 2017), nucleus accumbens (Darvish-Ghane et al., 2016) and subicular neurons (Behr et al., 2000), where 100 μM DA decreased the frequency of mEPSCs. DA did not affect kinetics properties such as the rise or decay time of sEPSCs and mEPSCs. This further supports the presynaptic effect of DA. Considering that our experiments are limited to the recording of sEPSCs and mEPSCs, in our future work, we will strengthen the findings by paired-pulse experiments and try to evoke electrically EPSCs from the HVC-RA input and from the LMAN input to RA using two distinct stimulation electrodes and test whether presynaptic dopamine receptors regulate differentially both excitatory inputs. It would be an interesting scientific question.

D1Rs are expressed in pyramidal neurons in the prefrontal cortex of mammals (Smiley et al., 1994; Trantham-Davidson et al., 2004). Our previous work demonstrated that DA could modulate the excitability in PNs of RA by binding to D1R (Liao et al., 2013). D1R may be expressed in PNs and will need to be validated in future studies.

Pharmacologically, the synaptic actions of DA indicate that the receptor mediating the decrease in excitatory synaptic transmission is D1R. The D1R agonist, but not the D2R agonist mimicked the actions of DA, while the D1R antagonist but not the D2R antagonist inhibited the actions of DA. This indicated that the observed effects of DA on the synaptic transmission are the result of DA binding to D1R, although the roles of D1R and D2R are determined through only one concentration of agonist/antagonist. These results were similar to the results observed in the nucleus accumbens in young rats, where the effects of DA on evoked EPSCs were similar to the D1-like receptor agonist SKF 38393 and were antagonized by the D1-like receptor antagonist SCH 23390. However, the D2-like receptor agonist or the antagonist failed to mimic or block the action of DA (Zhang et al., 2014).

Our previous work demonstrated that DA could modulate excitability in PNs of RA (Liao et al., 2013). This suggested that DA could indeed influence information processing in the song system by altering the input-output functions of the song control system. The present study indicated that DA could modulate glutamatergic synaptic transmission in the RA PNs of adult zebra finches. This validates our previous work on the effect of DA at the synaptic level (Liao et al., 2013). In our previous work, DA increased the excitability of RA PNs, while in the present study, we observed that DA inhibited excitatory synapse transmission. The excitability of RA PNs is determined by both excitatory and inhibitory synaptic inputs in the RA neural network. We only investigated DA regulation on the excitatory synaptic transmission of RA PNs in this study. The effects of DA on the inhibitory synaptic transmission of RA PNs need to be investigated. Our results additionally suggest that DA inhibits excitatory synaptic transmission through a presynaptic mechanism. DA may regulate cell activity balance through excitatory-inhibitory regulation.

The RA is a sensorimotor nucleus and receives a direct projection from the HVC and LMAN (Mooney and Konishi, 1991). A series of studies have demonstrated that RA intrinsic circuitry controls premotor output to produce songs (Meng et al., 2017). When male finches sing directed songs, the DA level from the midbrain input to Area X increases (Kubikova and Kostál, 2010), making Area X neurons more excited (Ding and Perkel, 2002). Area X activation increases inhibitory outputs to the thalamus to acutely decrease LMAN activity (Kojima and Doupe, 2009). This result suggests a decrease in excitatory synaptic transmission of the LMAN to the RA. Also, RA receives DArgic projections from the midbrain. DA depresses the excitatory synaptic transmission of RA, which may include the excitatory synaptic transmission of LMAN to RA. LMAN-RA contributes to the plasticity of song (Kao et al., 2008), hence DA reducing the excitatory synaptic transmission of LMAN-RA contributes to producing a more stable song.

In conclusion, the present study demonstrated that DA could inhibit excitatory synaptic transmission. D1R plays a significant role in regulating the excitatory synaptic transmission of RA PNs. Our results provide direct evidence to understand the mechanism of DA signal modulation on excitatory synaptic transmission of RA PNs.

## Data Availability Statement

The datasets generated for this study are available on request to the corresponding author.

## Ethics Statement

The animal study was reviewed and approved by Institutional Animal Care and Use Committee of Jiangxi Science and Technology Normal University.

## Author Contributions

SW, SL, QW, and LY performed the experiments. SW, SL, and WM designed the experiments and wrote the manuscript. QW, YS, and DL analyzed the data, WM contributed reagents, materials and analytical tools.

## Conflict of Interest

The authors declare that the research was conducted in the absence of any commercial or financial relationships that could be construed as a potential conflict of interest.
